# CD38 Drives Cellular Senescence and Ischemia-Reperfusion Injury in Donor Lungs

**DOI:** 10.21203/rs.3.rs-10988445/v1

**Published:** 2026-10-21

**Authors:** Dhiaeddine Djabri, Tony Boualoy, Jung Kim, Yonggyu Lee, Natalia Vanegas, Doug Gouchoe, Lorena Rosas, Xi Wang, Yuke Hou, Mahnoor Hamid, Quin Ma, Brenda F. Reader, Sylvester Black, Ana Mora, Mauricio Rojas, Bryan Whitson

## Abstract

Cellular senescence increases susceptibility to tissue injury and impairs reparative capacity, yet its role in lung transplantation-associated ischemia-reperfusion injury (IRI) and graft dysfunction remains poorly defined. Because senescence-associated signaling is intricately linked to metabolic dysfunction, we investigated the contribution of Cluster of Differentiation 38 (CD38), a key regulator of NAD⁺ homeostasis, to senescence-associated lung inflammation and injury. In lungs procured from human donors, CD38 expression increased with donor age and ischemic stress, particularly in donation after circulatory death (DCD) allografts, and was associated with senescence-associated secretory phenotype (SASP) signaling. In a murine hilar clamp lung IRI model, genetic deletion or pharmacologic inhibition of CD38 preserved NAD⁺ levels, attenuated SASP-associated inflammatory mediators, reduced histologic lung injury, and improved pulmonary mechanics. Bronchoalveolar lavage fluid and plasma analyses demonstrated that CD38 deletion or inhibition reduced inflammatory cytokines and cellular injury markers. In human umbilical vein endothelial cells cultures, CD38 knockdown attenuated oxidative stress-induced senescence and cellular injury markers, supporting a direct endothelial mechanism. Collectively, these findings position CD38 as a central mediator linking donor aging and ischemic stress to SASP-driven inflammatory injury and suggest that peri-transplant CD38 targeting may mitigate IRI, preserve allograft quality, and expand the donor lung pool.

